# Olecranon With Concomitant Radial Head Fracture: A Case Series of Fifteen Patients

**DOI:** 10.3389/fsurg.2022.838948

**Published:** 2022-05-03

**Authors:** Konstantinos Ditsios, Charalampos Pitsilos, Triantafyllos Katsimentzas, Panagiotis Konstantinou, Panagiotis Christidis, Pericles Papadopoulos

**Affiliations:** ^1^2nd Academic Department of Orthopaedic Surgery, General Hospital of Thessaloniki “G. Gennimatas”, Aristotle University of Thessaloniki, Thessaloniki, Greece; ^2^Department of Orthopaedic Surgery, General Hospital of Katerini, Katerini, Greece

**Keywords:** olecranon fracture, radial head fracture, elbow fracture, complex trauma, case-series

## Abstract

**Introduction:**

Simultaneous olecranon and radial head fractures are rare injuries and due to this factor, the outcome of the selected therapy is not widely studied. The aim of this study is to report and evaluate the functional outcome of the surgical treatment of simultaneous olecranon and radial head fractures.

**Materials and Methods:**

This is a retrospective study of fifteen patients with concomitant olecranon and radial head fractures presenting to our orthopedic department between 2015 and 2020. Olecranon fractures were classified by Mayo classification and radial head fractures by Mason classification and were managed appropriately. Main outcome measures include range of elbow extension-flexion, pronation-supination, Broberg and Morrey rating system score, and quickDASH score.

**Results:**

Our study included 6 females and 9 males with a mean age of 50 (r, 25–73). The mean of follow-up was 31 months (r, 3–51). Olecranon fractures were fixed with tension band with K-Wires or intramedullary compression screw or locking plate. Radial head fractures were fixed with headless compression screws or mini plate or replaced (radial head arthroplasty). Postoperatively, an average 115° extension-flexion arc and 135° pronation-supination arc was noted. The mean Broberg and Morrey rating system score was 78 and the mean quick DASH score was 25, indicating a good result. Two cases of heterotopic ossification were present and no nonunion was noted.

**Conclusion:**

Surgical management of concomitant olecranon and radial head fractures with appropriate technique can result in the restoration of a functional movement arc and a satisfactory outcome.

## Introduction

Fractures around the elbow joint need more meticulous care than other articular injuries, because of the multiple articulations involved in the elbow's normal motion, predisposing it to post-traumatic stiffness (1, 2). Complex fractures of proximal ulna and radius represent some of the most difficult fractures to treat, even for experienced orthopedic surgeons. Open anatomic reduction of the articular surface and stable internal fixation is the key to an effective treatment, as this approach enables early postoperative mobilization, which is of great importance (3).

There are some basic injuries of the elbow that must be recognized since each of them requires special treatment and affects the functional outcome. These injuries are ulnar fracture, radial head fracture, radiohumeral dislocation, ulnohumeral dislocation, proximal and distal radioulnar dislocation, interosseus membrane lesion, medial and lateral collateral ligament injury, and coronoid process fracture (4).

The precise classification of each injury and the personalization of each patient's injury are essential for optimal result. The olecranon fractures and the radial head fractures follow the Mayo clinic classification system (2, 5, 6) and the modified Mason classification (7–10), respectively. Regardless of the classification, the treatment strategy of this complex elbow injury pattern is not well defined and so are its outcomes. Case series with isolated olecranon fractures associated with radial head fractures are limited and outdated (11–13). The largest study involved 22 patients and was conducted approximately 25 years ago (13).

The objective of this study is to investigate the outcome measures of concomitant olecranon and radial head fractures treated operatively, based on a retrospective case-series of fifteen adult patients.

## Materials and Methods

We retrospectively reviewed the surgical treatment of olecranon fractures with concomitant radial head fracture analyzed in a case series of fifteen patients between 2015 and 2020. All surgeries were performed by the team of the same upper extremity orthopedic surgeon in the same hospital. A written informed consent was obtained from the patient and the institutional review board of our hospital approved this report. The following case reports are presented in accordance with Surgical CAse REport (SCARE) guidelines (14). This is a Level IV evidence study.

### Types of Patients

Patients aged 18 or older, of any gender or race, diagnosed with only olecranon fracture associated with radial head or neck fracture. These injuries could have been isolated or part of multiple trauma cases. Additionally, these patients should have been treated operatively and not have been lost during follow-up.

### Preoperative Procedures

**Figure 1:**
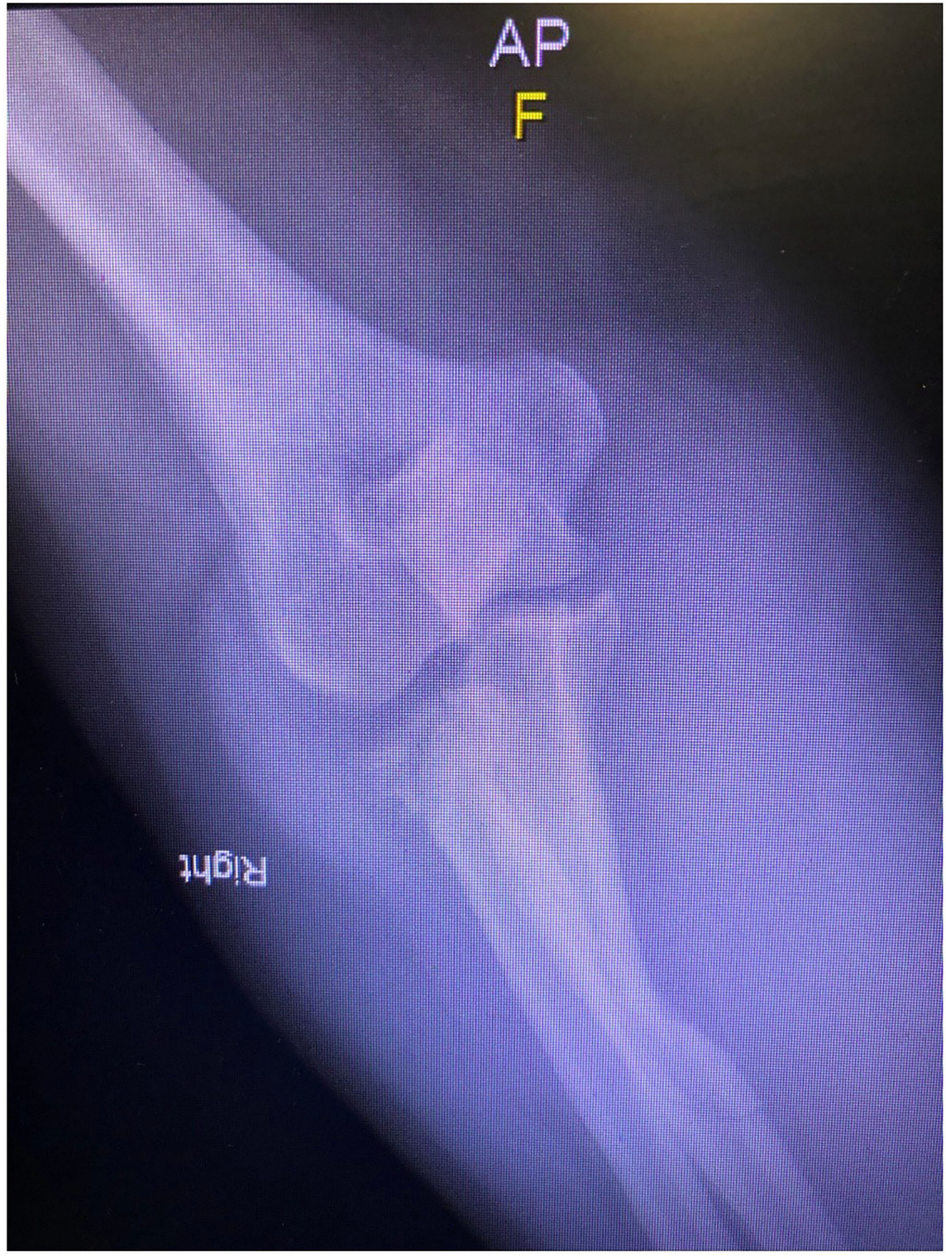
Preoperative elbow AP view of a complex elbow trauma.

**Figure 2:**
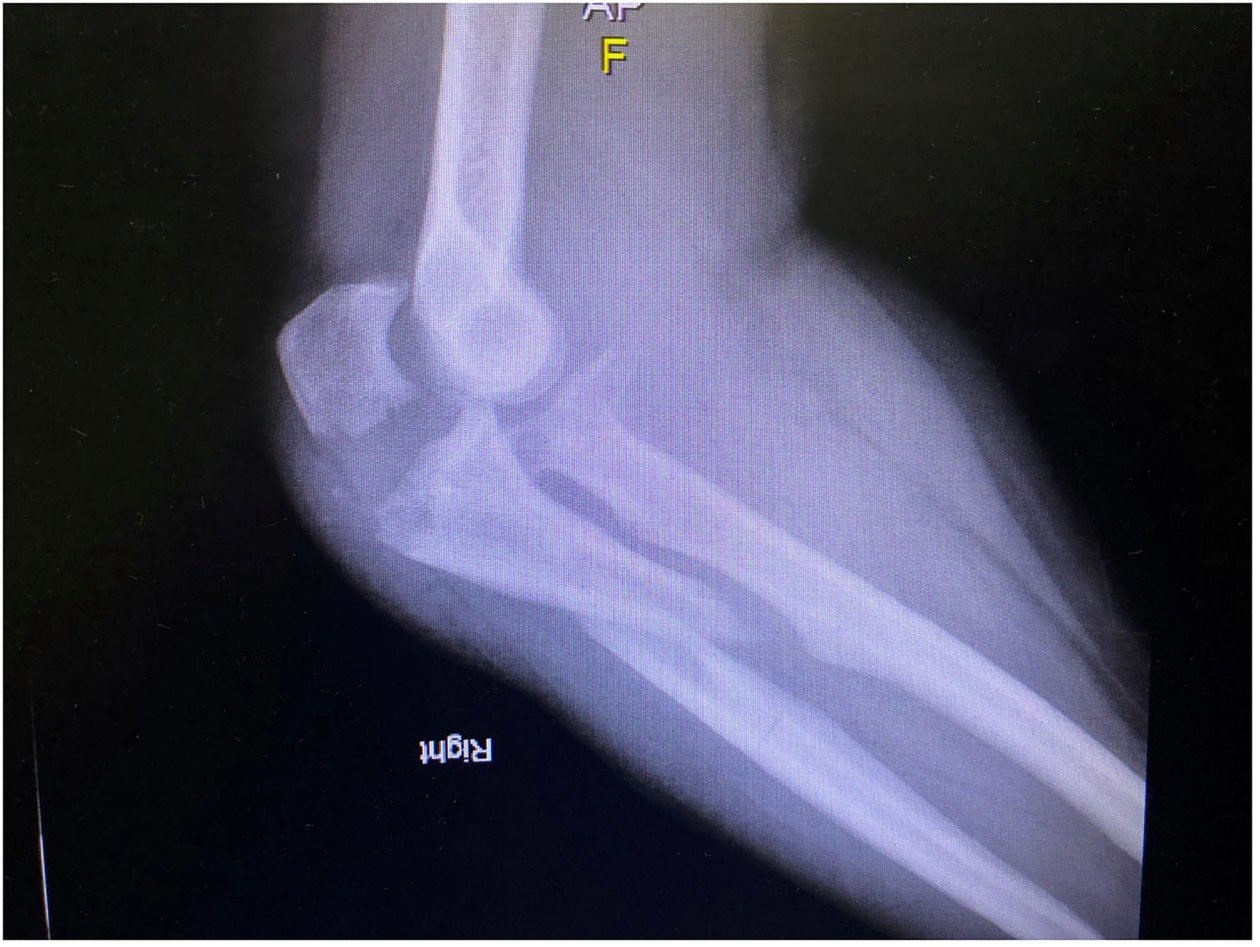
Preoperative elbow lateral view of a complex elbow trauma.

Preoperative imaging modalities include elbow anteroposterior (AP) and lateral x-rays (Figures 1, 2), and CT scanning with 3D reconstruction in all patients. Olecranon fractures were classified by Mayo classification as type II displaced-stable and type III displaced unstable. Letter “A” indicates simple fracture and letter “B” indicates comminution. Radial head fractures were classified by Mason classification as type II displaced and type III comminuted and displaced.

### Surgical Approach

All operations were performed under general anesthesia, the patient was placed in supine position with the affected extremity in a cross-chest position and a tourniquet was inflated. All fractures were exposed through the posterolateral Boyd's approach. This approach is useful to reach both proximal ulna and radial head *via one* skin incision, which starts laterally at the supracondylar ridge at the level of the superior border of the forearm and continue slightly more lateral crossing directly over the lateral epicondyle. It curves distally over the lateral aspect of the tip of the olecranon and continues along the subcutaneous border of the ulna. The ulna is approached behind the anconeus proximally and extensor carpi ulnaris more distally. Radial head is approached by reflecting anconeus muscle anterolaterally after incising its ulnar insertion and then by detaching the supinator near its ulnar origin.

**Figure 3:**
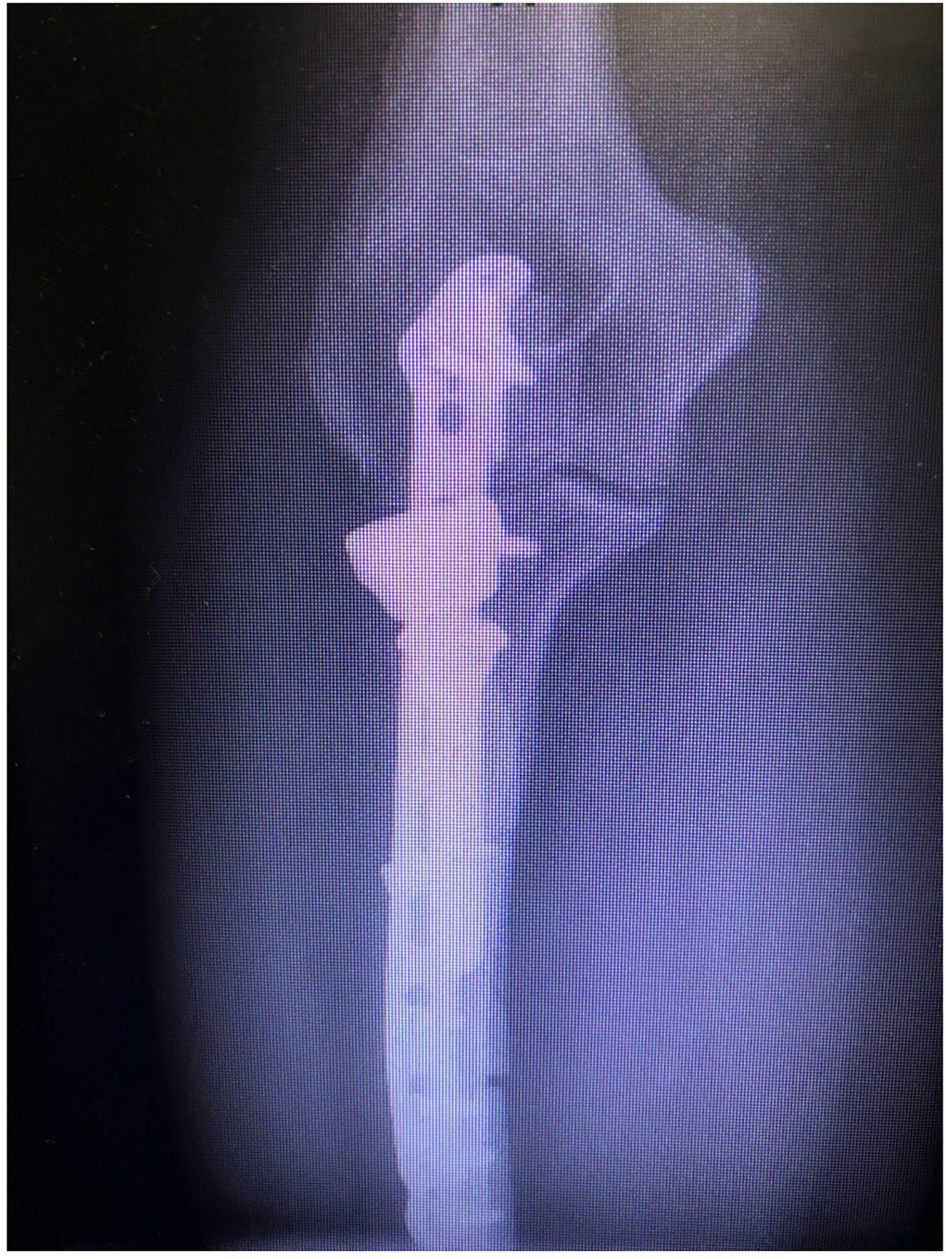
Post-op AP view (osteosynthesis of the ulna with proximally contoured locking plate and radial head arthroplasty).

**Figure 4:**
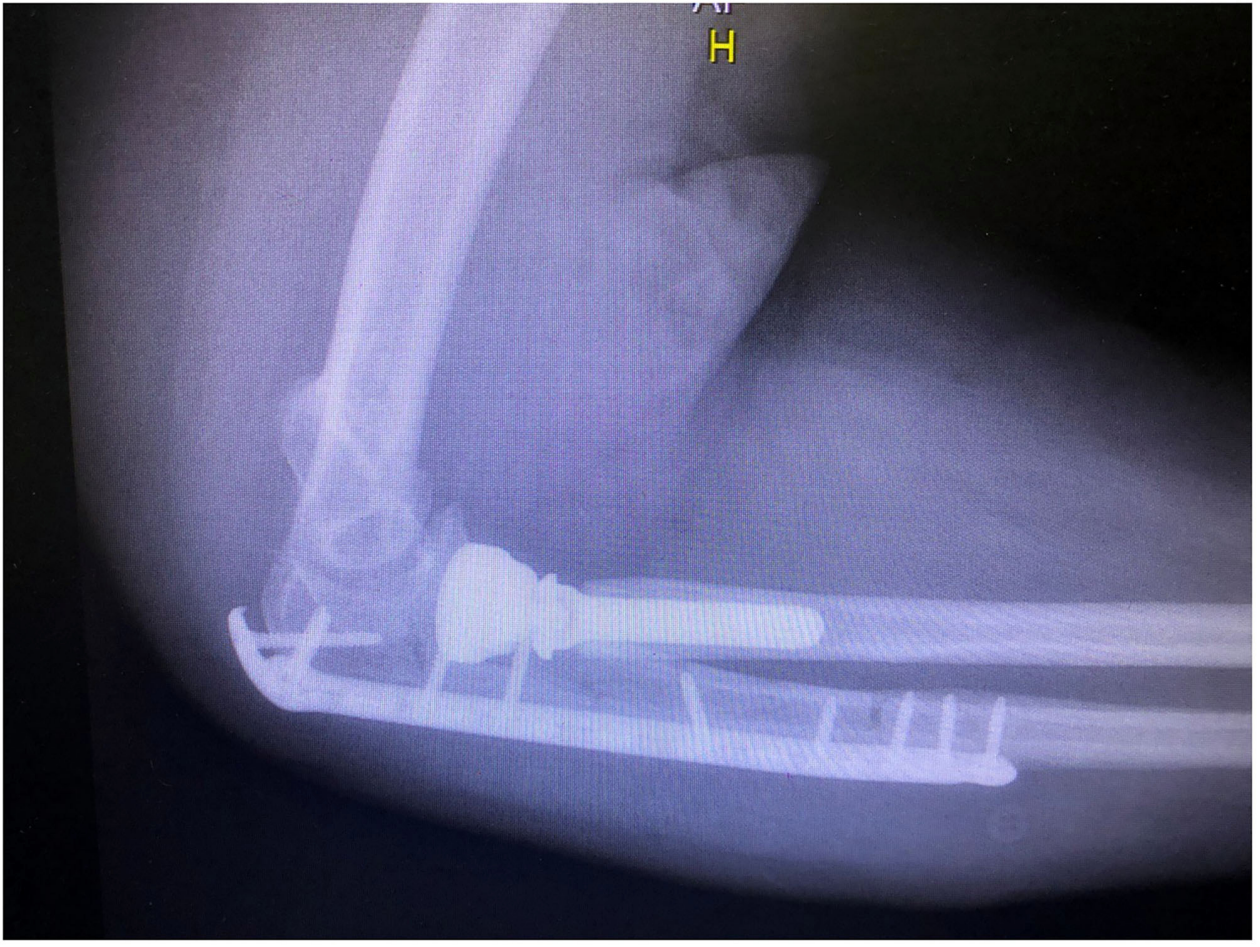
Post-op lateral view (osteosynthesis of the ulna with proximally contoured locking plate and radial head arthroplasty).

Olecranon fracture fixation technique included tension band wiring with K-wires (4) or intramedullary compression screw (1) or proximally contoured locking compression plate (LCP) 3.5 mm (10) (Figures 3, 4). The radial fracture was fixed by Herbert headless compression screws (3), mini plate (2), and osteosynthesis or radial head arthroplasty (10) (Figures 3, 4), depending on the fracture pattern. In three cases, where a coronoid fracture presented, it was fixed by a cortical screw through the olecranon plate. In every case as soon as the osteosynthesis was done the surgeon checked for elbow stability.

### Post-Operation Procedures

In all patients, postoperative x-rays of the elbow were obtained. For antimicrobial prophylaxis, a second-generation cephalosporin (cefoxitin) was used intravenously, 1 gr injected 30 min before surgery and 1 gr injected every 8 h for the first 2 postoperative days.

The post-surgery rehabilitation protocols alter significantly among the patients, depending on the quality of the bone seen intraoperatively and the presence of a coronoid fracture, while early mobilization was the main goal. A posterior long arm splint was placed to immobilize the elbow joint for the first 2 weeks, aiding the edema absorption. Then sutures were removed, and a functional elbow cast replaced the splint allowing early passive motion at first, followed by the active motion of flexion, extension, pronation, and supination as soon as possible. Loading was prevented for 6–8 weeks.

### Outcome Measures

Patients were evaluated with the Broberg and Morrey rating system which incorporates numerical values for motion, strength, stability, and pain as criteria for evaluation of the elbow (15). The quickDash score was also used, which measures the physical function and symptoms while doing eleven everyday activities (16). The baseline function of the uninjured extremity was not examined.

X-rays were obtained for the evaluation of fracture union, or the presence of adverse events, such as malunion, heterotopic ossification etc. Imaging study included, elbow anterioposterior and lateral x-ray at 1, 3, and 6 months post-operatively and once annually since then. The range of motion was determined in terms of elbow flexion, elbow extension, forearm pronation, and forearm supination with a goniometer, at the time of this review. Heterotopic ossification of the elbow is graded by the Hastings and Graham classification based on its severity and functional limitation (17). Finally, fracture union was defined as the bridging bone on anteroposterior and lateral radiographs.

### Statistical Analysis

Continuous variables were expressed by the arithmetic mean and standard deviation (m ± sd, range). Qualitative variables, categorical or ordinal, are presented as numbers and percentages per 100. The confidence interval was set at 95% which means that the differences between the groups were considered statistically significant when *p* < 0.05. The statistical analysis of the results was performed using the statistical program Jamovi Version 1. 6. 18.0.

## Results

### Patients' Descriptive Characteristics

In total, fifteen patients with combined olecranon and radial head fractures were included in this study (9 males, 6 females). Median age at the time of operation was 49.9 ± 13.5 years (range 25–73). None of these fractures was an open fracture. No nerve injury was noted or signs of dislocation. In nine cases the dominant hand was involved. In five cases, the injury was a result of high energy trauma and in the rest of the 10 cases, it happened after a fall on an outstretched hand. The average follow-up period was 31 months (range 3–51). The mean time from the injury to surgery was 1 day.

### Fixation Strategies

Olecranon fracture was classified as Mayo type IIA in 5 patients, type IIB in 5 patients, type IIIA in 3 patients, and type IIIB in 2 patients. Radial head fractures were classified as Mason type II in 9 patients and type III in 6 patients. Combined treatment included tension band wiring with intramedullary compression screw for olecranon fracture and fixation of radial head fracture with Herbert screw in 1 patient, tension band wiring with K-Wires for olecranon fracture and fixation of radial head fracture with Herbert screw in 2 patients, tension band wiring with K-Wires for olecranon fracture and fixation of radial head fracture with the mini plate in 2 patients, tension band wiring with K-Wires for olecranon fracture and radial head arthroplasty in 1 patient, fixation of olecranon fracture with locking plate and fixation of radial head fracture with Herbert screw in 4 patients, fixation of olecranon fracture with locking plate and fixation of radial head fracture with mini plate in 1 patient and fixation of olecranon fracture with locking plate and radial head arthroplasty in 4 patients (Table 1). In 3 cases there was a coronoid fracture which was stabilized with screws.

**Table 1:** Patient's data.

**No**	**Age**	**Sex**	**MoI**	**Olecranon fracture type (MAYO)**	**Radial head fracture type (MASON)**	**Broberg-Morrey Score**	**Quick-DASH Score**	**Extension-Flexion arc**	**Pronation-Supination arc**	**Type of surgery (Olecrano fr. + Radial fr.)**
1	43	M	FOOSH	IIIA	III	82	26	125	150	Locking plate + RHA
2	41	M	HE	IIB	III	73	30	120	147	Locking plate + Mini plate
3	53	M	FOOSH	IIA	II	81	26	125	150	TB (ICS) + Herbert screws
4	62	F	FOOSH	IIB	III	64	38	105	150	TB (K-W) + RHA
5	55	M	FOOSH	IIIA	II	81	18	110	135	Locking plate + Herbert screw
6	25	F	HE	IIA	II	95	12	125	160	TB (K-W) + Herbert screws
7	71	F	FOOSH	IIB	II	67	42	100	130	Locking plate + RHA
8	73	M	FOOSH	IIA	II	66	39	105	125	Locking plate + Herbert screws
9	45	M	HE	IIA	III	85	17	115	160	TB (K-W) + Mini plate
10	51	F	FOOSH	IIB	III	83	18	105	140	Locking plate + RHA
11	35	M	HE	IIIB	II	77	20	105	155	Locking plate + Herbert screws
12	37	F	HE	IIA	II	94	13	120	155	TB (K-W) + Herbert screw
13	64	M	FOOSH	IIIA	II	69	34	120	140	TB (K-W) + Mini plate
14	50	M	FOOSH	IIIB	III	71	27	115	130	Locking plate + RHA
15	44	F	FOOSH	IIB	II	88	15	130	148	Locking plate + Herbert screws

*M, male; F, female; MoI, Mechanism of Injury; FOOSH, Fall On Out-Stretched Hand; HE, High Energy trauma; RHA, Radial Head Arthroplasty; TB (ICS), Tension Band wiring with Intramedullary Compression Screw; TB (K-W), Tension Band wiring with K-Wires; Letter “A” indicates simple fracture and letter “B” indicates comminution*.

### Complications

Complete union was achieved in all cases during the follow up period. There were noted no infections or nerve palsies in the early postoperative period. The reduction achieved during surgery was preserved in all cases and there were no implant failures. Two cases of heterotopic ossification were noted, one graded as Class IIA and one as Class IIB according to Hastings and Graham classification, with minor effect on the functional outcome.

### Outcome Measures

The average degrees of elbow motion, at the time of this review, were 15° extension, 130° flexion (average 115.0 ± 9.4° extension-flexion arc), 70° pronation, and 65° supination (average 145.0 ± 11.1° pronation-supination arc). The mean Broberg and Morrey rating system and quickDASH score was 78.4 ± 9.9 (range 64–95) and 25.0 ± 9.9 (range 12–42) points respectively, indicating a good result (Table 1).

## Discussion

In this study, we present the outcomes of fifteen patients presented with concomitant olecranon and radial head fracture. We chose olecranon and radial head fracture management based on Mayo and Mason classification respectively. The outcome reflected in Broberg and Morrey rating system and quickDASH score seems to be at least satisfying.

Olecranon and radial head fractures are common injuries of the upper extremity when presented separately and they have been studied and analyzed in depth. On the other hand, simultaneous fractures compromise an unusual injury pattern and the proper treatment, and its outcomes have not been deeply investigated. Due to lack of evidence-based instructions about the management, we dealt with each fracture as a unique entity and tried to preserve elbow joint stability and range of motion. Olecranon fractures were treated by placing 5 tension band wires with K-wires, 1 tension band wire with intramedullary compression screw and 9 proximally contoured LCP 3.5 mm plates. We treated radial head or neck fracture performing 10 open reductions and internal fixations, 7 with Herbert headless screws, and 3 with mini plates, and 5 radial head arthroplasties. Newer fixation techniques alleviated complications of non-union or delayed union, which according to Heim et al. were observed with comminution of these fractures (13). No ligamentous laxity was observed, which would require further restoration as ligamentous stability is paramount for optimal post-operative outcomes (12).

While separate olecranon and radial head fractures have been studied and classified by many different authors, there is lack of classifications of this specific fracture pattern Only two attempts were found searching PubMed database. AO classifies this as type 2R1B/2U1B, 2R1B/2U1C, 2R1C/2U1B and 2R1C/2U1C based on fracture location and comminution (18). Giannicola et al. (4) proposed a classification called Proximal Ulnar and Radial fracture-dislocation Comprehensive Classification System (PURCCS) for proximal ulnar and radial fracture-dislocation, defining olecranon and simultaneous radial head fracture pattern as type 1CI or 1CIII and 3CI or 3CIII when an additional coronoid fracture is present (4).

Moreover, some authors include this injury in Monteggia-equivalent lesion that encompass a wide spectrum of fractures of the forearm and elbow associated with dislocations, subluxations, and ligamentous lesions (10, 19), while other not (20, 21). We disagree with this characterization, because Monteggia lesions describe proximal ulnar fractures with dislocation of proximal radioulnar articulation, and the latter does not happen when the olecranon fracture is accompanied by a radial head fracture. On the contrary, radiocapitellar dislocation may be present in both injury patterns, but still, not always. Monteggia-like lesions have been studied in pediatric population, too. Gokkus et al. published an article about olecranon fractures associated with radial neck fractures in the pediatric population, considering them as Monteggia-equivalent (22). They searched PubMed database and concluded that this injury pattern is rare in children, too (23). Cepelík et al. accepted as Monteggia-like lesions in children fractures of ulna in any level conjoined with a displaced fracture of the proximal radius, but not undisplaced fracture patterns (24). In conclusion, there is discordance about both classification and description of concomitant olecranon and radial head fracture as Monteggia-equivalent injury.

A major postoperative goal following trauma to the elbow, is to regain a satisfactory range of motion. In their biomechanical analysis, Morrey et al., pointed out that most daily activities can be done with 100 of elbow flexion (from 30 to 130) and 100 of forearm rotation (50 of pronation and 50 of supination) (2). In our clinical examination, the arc of motion averaged 115 of flexion and 145 of forearm rotation, showing that “Morrey's rule” was accomplished in all our patients. Our results agreed with Perry and Tessier that demonstrated an average arc of pronation/supination of 124° and an average arc of flexion/extension of 113° in six patients with similar injury pattern (11). In our study, we used the Broberg and Morrey rating system as the basic evaluation instrument assisted by the quickDASH score, and we concluded that the functional outcome of our surgical intervention was more than satisfactory in all cases. In Broberg and Morrey rating system the lowest score was 64, which corresponds to fair outcome, whereas 8 patients had a score of 80 and more, showing a good to excellent result. In respect to the quickDASH score, results ranged from 12 to 42 conforming to an over the average functional outcome. Our approach, treatment strategy and postoperative rehabilitation were effective enough to result, at least, in a satisfactory functional outcome, which is the main goal of fracture treatment.

This positive outcome is owed to the lack of postoperative complications, short immobilization period, and lack of ligamentous instability. In our case series two patients developed HO, one graded as Class IIA, with limitation of flexion/extension arch and one as Class IIB, with limitation of pronation/supination arch. The functional deficit was not of much importance and any intervention was not decided. Other than this complication, none was identified in postoperative follow-up. Chen et al. concluded that prolonged immobilization strategies are of no benefit in a similar case, instead he advocated for short periods of immobilization followed by accelerated rehabilitation (12). Comminution of these fractures may cause delayed union or non-union, according to Heim et al., however we did not observe these complications in our patients (13).

The present study has several limitations, the most important is the small sample size and its retrospective nature. Also, the included patients presented with a great spectrum of different fracture types making a detailed comparison between specific fracture patterns impossible. The short follow up period of some patients, may hind future complications such as heterotopic ossification and osteoarthritis.

Simultaneous olecranon and radial head fractures are rare injuries and due to this factor, the outcome of the selected therapy is not widely studied and cannot be predicted. However, surgical management of combined olecranon and radial head fractures with correct technique can result in the restoration of a functional movement arc and a satisfactory outcome. Of course, the small number of cases is a limitation to reach a reliable conclusion and, for this reason, larger studies are needed to build a treatment strategy for patients with combined olecranon and radial head fractures.

## Data Availability Statement

The original contributions presented in the study are included in the article/supplementary material, further inquiries can be directed to the corresponding author/s.

## Ethics Statement

Ethical review and approval was not required for the study on human participants in accordance with the local legislation and institutional requirements. The patients/participants provided their written informed consent to participate in this study.

## Author Contributions

KD, CP, TK, and PK conceived the study's objective, performed data collection and extraction, performed the operations, and were responsible for the post-operative course of the patient. All aforementioned authors along with PC and PP wrote the initial draft. All authors made substantial contributions and reviewed the document carefully prior to submission. All authors contributed to the article and approved the submitted version.

## Conflict of Interest

The authors declare that the research was conducted in the absence of any commercial or financial relationships that could be construed as a potential conflict of interest.

## Publisher's Note

All claims expressed in this article are solely those of the authors and do not necessarily represent those of their affiliated organizations, or those of the publisher, the editors and the reviewers. Any product that may be evaluated in this article, or claim that may be made by its manufacturer, is not guaranteed or endorsed by the publisher.
